# Central nervous system involvement of systemic ALK-positive histiocytosis with *KIF5B-ALK* fusion

**DOI:** 10.1016/j.radcr.2022.07.072

**Published:** 2022-08-10

**Authors:** Yuzuna Aoki, Masayuki Maeda, Seiya Kishi, Ryota Kogue, Fumine Tanaka, Maki Umino, Mami Takeoka, Ryo Hanaki, Junya Hirayama, Hiroto Yuasa, Hiroshi Imai, Masahiro Hirayama, Hajime Sakuma

**Affiliations:** a Department of Radiology, Mie University School of Medicine, Tsu, Mie, Japan; b Department of Neuroradiology, Mie University School of Medicine, 2-174 Edobashi, Tsu 514-8507, Mie, Japan; c Department of Pediatrics, Mie University School of Medicine, Tsu, Mie, Japan; d Pathology Division, Mie University Hospital, Tsu, Mie, Japan

**Keywords:** ALK-positive histiocytosis, ALK gene fusion, CT, MRI, FDG-PET

## Abstract

ALK-positive histiocytosis is an emerging histiocytic entity that can involve a single organ or multiple organs. This disease frequently involves the central nervous system, and the importance of immunohistochemical and genetic analyses is emphasized for the accurate diagnosis of this rare entity. However, radiological findings of this disease have not been sufficiently described. Here, we report a case of a 3-year-old boy with ALK-positive histiocytosis with systemic masses that was identified to harbor *KIF5B-ALK* gene fusion.

## Introduction

ALK-positive histiocytosis is an emerging subtype of histiocytic neoplasm first described in 2008 in 3 infants with multisystemic disease [1]. Among the 3 cases, one case showed a *TPM3-ALK* gene rearrangement. After this report, several case reports or case series about ALK-positive histiocytosis have been described and piled up, confirming that it is a new histiocytic entity [2], [3], [4], [5], [6]. These reports demonstrated that this disease frequently occurs in children but also in adults, either with a single organ or multisystemic disease. Notably, frequent incidence of *KIF5B-ALK* gene fusions was reported [3–6], indicating that ALK inhibition is a promising therapeutic option for this entity. Thus, it is important to identify this entity by using an integrated histologic and genetic approach for the diagnosis. On the other hand, radiological findings on this entity have not been sufficiently described. Accordingly, here we report on the imaging features in a case with systemic ALK-positive histiocytosis with *KIF5B-ALK* fusion involving central nervous system (CNS).

## Case presentation

A 3-year-old boy presented for 3 weeks with left nasal obstruction, a lump in the right inner canthus, and hyperemia of the left eye. Physical examination showed no particular findings, including skin. Blood tests showed mild anemia, mild thrombocytosis, and a mildly elevated C-reactive protein. He had no measurable liver dysfunction or significant coagulation abnormality. Cerebrospinal fluid analysis was normal.

**Fig. 1:**
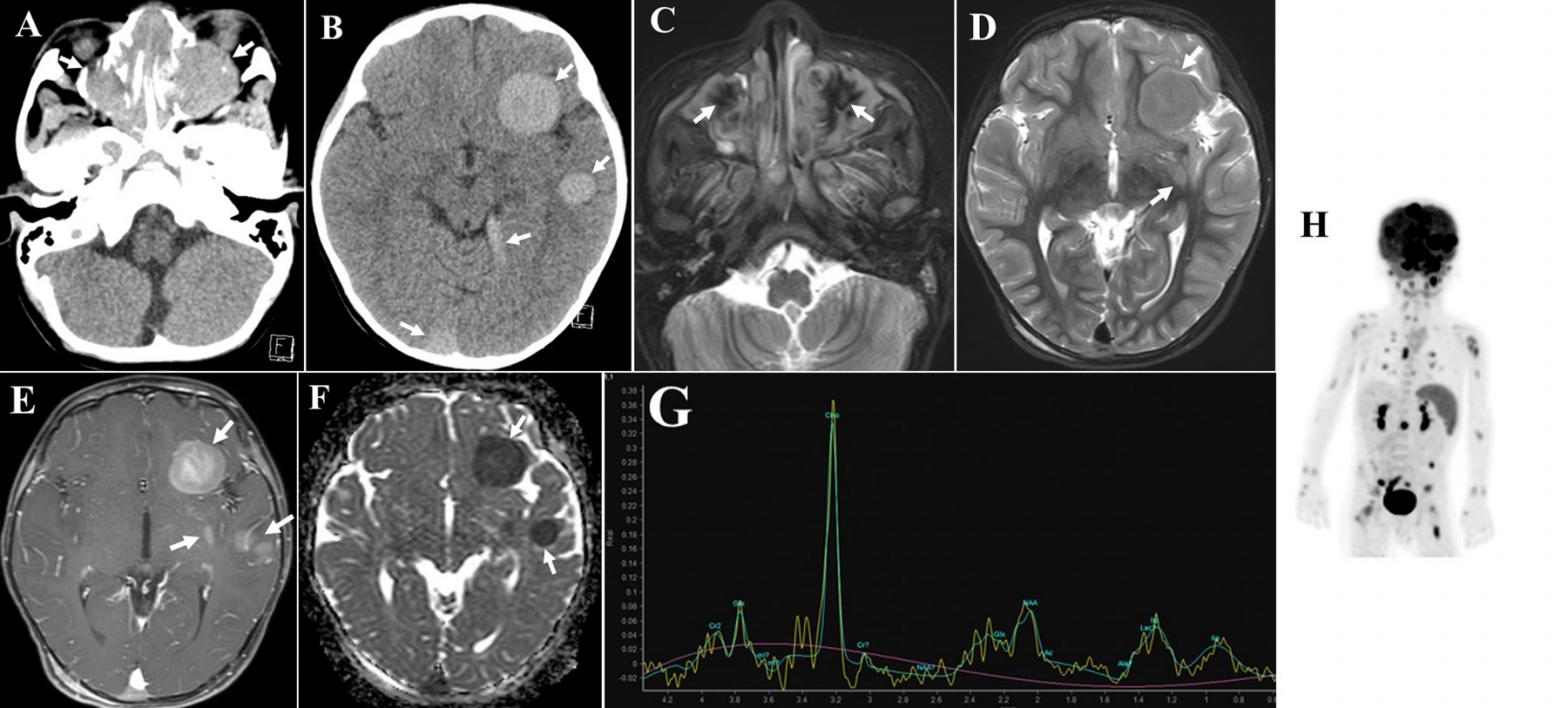
Plain CT images show bilateral nasal and paranasal soft tissue masses with bony destruction (A: arrows) and multiple discrete hyperdense masses in either intra-axial or extra-axial brain (B: arrows). STIR images show the low signal intensity in the middle of maxillary sinus lesions (C: arrows) and isointense masses with no edema in the brain (D: arrows). Contrast-enhanced T1-weighted image shows multiple homogeneously enhancing masses (E: arrows). ADC map shows restricted diffusion of the masses (F: arrows). MRS shows increased choline peak and the presence of lipid and lactate (G). FDG-PET shows multiple uptakes of the lesions (H).

Head CT showed the masses of the bilateral nasal/paranasal sinuses and multiple hyperdense masses in the brain (Figs. 1A and B). The masses were well-defined and smooth, with osteolytic changes in the paranasal sinuses. No obvious calcification was observed. MRI showed nasal/paranasal sinus masses and multiple intracranial masses with intra-axial and extra-axial locations. Most intra-axial brain masses were iso-intensity to the normal cortex on T1WI and STIR, and hyperintensity on DWI. The masses in the bilateral nasal/paranasal sinuses and extra-axial brain showed marked hypointensity in the middle of the lesions on STIR (Fig. 1C). The masses had no edema, and were enhanced uniformly (Figs. 1D and E). ADC value of the intra-axial brain masses was markedly decreased (mean 0.57 × 10^−3^ mm^2^/s; Fig. 1F). MR spectroscopy showed high Cho peak and the presence of Lac/Lip peak (Fig. 1G). Pseudocontinuous arterial spin labeling images showed almost equal tumor blood flow to that of normal brain. Amide proton transfer (APT) imaging showed APT-related signal intensity of the masses was isointense to that of the normal brain. FDG-PET showed the intracranial and paranasal masses had abnormal uptake with a SUVmax of 10.7, and it also showed several other abnormal uptakes in the lungs, pancreas, spleen, appendix, lymph nodes, and bones (Fig. 1H).

**Fig. 2:**
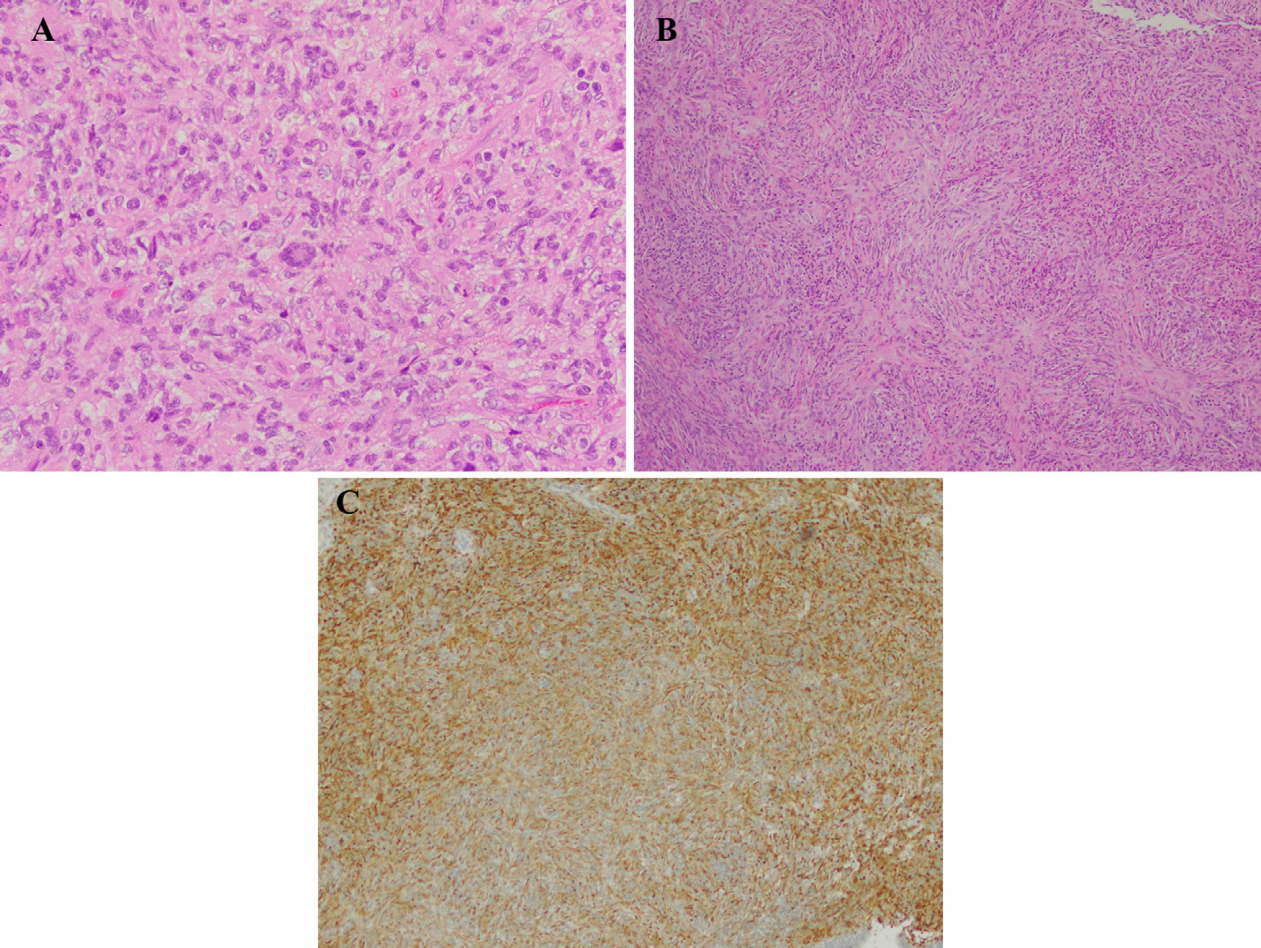
Histopathological examination of the resected specimen. Hematoxylin and eosin staining show Touton giant cell, histiocyte-like cells, and spindle-shaped cells (A), areas of massive fibrosis (B). Immunohistochemical staining shows positive for ALK (5A4) (C).

He underwent nasal tumor biopsy for pathologic diagnosis. The biopsy of the sinus tumor showed diffuse proliferation of histiocyte-like cells with round nuclei with fine nuclear chromatin and pale eosinophilic cytoplasm, accompanied by eosinophils, plasma cells, and lymphocytes. Cells with curved, club-shaped nuclei, foam cells, Touton giant cell, foreign body giant cells, and spindle-shaped cells proliferating in bundles were also seen (Fig. 2A). These findings suggested the diagnosis of histiocytosis, particularly suspicious of juvenile xanthogranuloma and Langerhans cell histiocytosis. Massive fibrosis was also found in the biopsy specimen (Fig. 2B). The proliferating cells, including spindle-shaped cells, were CD163 and vimentin positive, while CD1a, S-100 protein, CD68 (PGM1), CD30, myeloperoxidase, cytokeratin (AE1/AE3), and α-SMA negative. Immunostaining for ALK1 was weakly positive, but ALK (5A4) was positive in the cytoplasm (Fig. 2C). Break-apart fluorescence in situ hybridization, the assay for detecting ALK rearrangements, showed a split signal in this tumor. Targeted next-generation sequencing (FoundationOne CDx, Foundation Medicine Inc., Cambridge, MA), identified in-frame *KIF5B-ALK* gene fusion. Considering these results, we diagnosed this case ALK-positive histiocytosis with *KIF5B-ALK* gene fusion.

## Discussion

We demonstrated detailed radiological findings of a rare case of ALK-positive histiocytosis that was genetically confirmed to have *KIF5B-ALK* gene fusion. ALK-positive histiocytosis is frequently associated with *KIF5B-ALK* fusions and is characterized by frequent neurologic involvement [6]. Although the number of reports about this distinct entity has increased since its introduction in 2008, the descriptions have focused on the genetic features or treatments such as ALK inhibitors, whereas the disease has not been described in the radiological literature. This case report focused on radiologic findings of this rare entity.

Although previous investigators mostly reported a case or case series about ALK-positive histiocytosis, Kemps et al. recently reported the largest study of ALK-positive histiocytosis, with detailed clinicopathologic data of 39 cases (child 79%), including 37 cases with confirmed ALK rearrangements [6]. According to this report, the clinical spectrum consisted of distinct clinical phenotype groups: infants with multisystem disease with liver and hematopoietic involvement as originally reported (6/39), patients with other multisystem disease (10/39), and patients with single-system disease (23/39). Particularly, 19 cases of the total cohort (49%) had CNS involvements. So far as known, there are reports of a total of 89 cases with ALK-positive histiocytosis that had ALK immunoreactivity and/or ALK rearrangement [6]. Of these, 27 cases had CNS involvement (30.3%). Considering the frequent involvement of CNS in this disease, radiologists should know more about this disease.

In our patient, FDG-PET showed multiple masses throughout the body with high uptake. Brain MRI showed multiple isointense masses with homogenously decreased ADC and diffuse enhancement. Therefore, we first suspected a hematologic malignancy such as leukemia based on the imaging findings. These MR imaging findings mimic those reported previously in juvenile xanthogranuloma [7]. Rounded to spindled, variably vacuolated numerous histiocytes with occasional lymphocytes and eosinophils might explain these findings. In this study, bilateral nasal/paranasal sinuses showed markedly low signal areas on STIR, which is an unusual finding for hematologic malignancy described above. In fact, the biopsy results showed dense fibrosis, suggesting that it explains markedly low signal intensity on STIR. Thus, markedly low signal areas within masses might be a clue to the diagnosis of histiocytosis, since it is occasionally seen in cases with head and neck histiocytosis [8]. Nevertheless, imaging findings of ALK-positive histiocytosis appear non-specific, and resemble those of Erdheim-Chester disease or juvenile xanthogranuloma, showing multisystemic or a single organ involvement [6].

In patients with ALK-positive histiocytosis with *KIF5B-ALK* fusions, robust and durable responses to ALK inhibitions were reported [4], [5], [6]. Despite the effective response to ALK inhibitions, it remains undefined whether ALK inhibitions should be implemented as first-line treatment or when disease is refractory to conventional therapies [6]. However, it is clinically valuable that ALK inhibitions can be a promising therapeutic option for this entity. Therefore, it is important to identify this entity by using an integrated histologic and genetic approach for the diagnosis and treatment.

## Conclusion

We presented a child case with ALK-positive histiocytosis with systemic masses that was identified to harbor *KIF5B-ALK* gene fusion. Brain MRI showed homogeneously restricted diffusion, lack of edema, homogeneous enhancement. Hypointense lesions on STIR may suggest the diagnosis of histiocytosis. Further investigation with larger number of cases is expected since ALK inhibitions are reportedly effective in this disease.

## Patient consent

Written informed consent for publication of the case report was obtained from the parents of the patient.
